# Tracking keratinocytes and melanocytes using carboxyfluorescein hydroxysuccinimidyl ester staining

**DOI:** 10.1371/journal.pone.0221878

**Published:** 2019-08-29

**Authors:** Susanna Lönnqvist, Johan P. E. Junker, Maria Sedell, Erika Nyman, Gunnar Kratz

**Affiliations:** 1 Division of Experimental Plastic Surgery, Department of Clinical and Experimental Medicine, Linköping University, Linköping, Sweden; 2 Center for Disaster Medicine and Traumatology, Department of Clinical and Experimental Medicine, Linköping University Hospital, Linköping, Sweden; 3 Department of Hand Surgery, Plastic Surgery and Burns, Linköping University Hospital, Linköping, Sweden; University of South Carolina, UNITED STATES

## Abstract

**Introduction:**

The treatment of burn wounds and hypopigmentation conditions often require autologous transplantation of keratinocytes and melanocytes. Tracking transplanted cells to ascertain their contribution to tissue recapitulation presents a challenge. This study demonstrates a methodology based on passive staining with carboxyfluorescein hydroxysuccinimidyl ester (CFSE) that enables localization of cells in tissue sections to investigate the fate of transplanted cells in wound re-epithelialisation.

**Methods:**

Viability and migration of CFSE-stained keratinocytes and melanocytes were investigated using viability staining and scratch assays, while proliferation of cells was measured using flow cytometry. In addition, CFSE-stained keratinocytes and melanocytes were transplanted to a human *ex vivo* wound model, either in suspension, or with the aid of macroporous gelatine microcarriers. Wounds were analysed seven, 14 and 21 days post transplantation using cryosectioning and fluorescence microscopy. Sections from wounds with transplanted co-cultured keratinocytes and melanocytes were stained for pancytokeratin to distinguish keratinocytes.

**Results:**

CFSE-staining of keratinocytes and melanocytes did not affect the viability, migration or proliferation of the cells. Transplanted cells were tracked in *ex vivo* wounds for 21 days, illustrating that the staining had no effect on wound re-epithelialisation. In conclusion, this study presents a novel application of CFSE-staining for tacking transplanted primary human keratinocytes and melanocytes.

## Introduction

Major traumatic loss of skin, particularly from burns, frequently require skin grafting for repair [1, 2]. In a large burn, donor sites are limited resulting in the need for skin graft expansion [2]. Using meshing [3] or mincing [4], or micrografting [5], skin grafts can be expanded. However, in cases of extreme skin loss, expansion using surgical methods may be inadequate. Green *et al*. developed methods for *in vitro* culture of keratinocytes, allowing an almost unlimited expansion potential [6, 7]. The resulting skin constructs, termed ‘Cultured Epidermal Autografts’ (CEA), are routinely used as an alternative to split-thickness skin grafts in the treatment of large defects [8]. Other epidermal cells in clinical use include autologous melanocytes for the treatment of hypopigmentation disorders (*ie* vitiligo) [9]. Moreover, co-transplantation of melanocytes and keratinocytes can be a successful strategy as some factors secreted by keratinocytes have been shown to sustain melanocyte growth [10].

When evaluating novel treatment methods based on autologous cell transplantation for burns or other non-healing wounds, it is often difficult to ascertain whether the transplanted cells contribute to the regenerative process. Most current protocols for labelling cells for subsequent tracking *in situ* rely on genetic modification, which is cumbersome and with varying efficiency and stability. Moreover, the safety of using these cells in a clinical context is questionable.

The non-fluorescent pro-dye 5(6) carboxyfluorescein-N-hydroxysuccinimidyl ester (CFSE) is a passively up-taken cell stain conventionally used for proliferation studies of lymphocytes and other blood cells [11]. Covalently coupled to mainly lysine residues the staining is inherited at cell division enabling proliferation studies with flow cytometry on account of the discrete steps of dye dilution in each mitotic generation. CFSE can be used in long term proliferation studies [12] and remains in keratinocytes for up to two weeks [13]. A study published by Cong *et al*. demonstrated the successful tracking of transplanted retinal pigment epithelium using CFSE in a rabbit model [14].

A previously described *ex vivo* wound healing model applies viable human full thickness skin samples with a standardised central deep dermal wound [15]. In the study, the authors demonstrate that wounds remain viable for up to four weeks in standard cell culture medium supplemented with 10% foetal calf serum, during which time the re-epithelialisation of the standardised wounds can be studied. This represents a highly controllable and standardized wound model based on healthy human tissue. Transplantation of cells (*ie* keratinocytes and melanocytes) can be studied using the *ex vivo* wound model, either with cells delivered in suspension or attached to macroporous gelatine microcarrier scaffolds. The microcarriers provide a large culture surface and have been shown to facilitate cell expansion and subsequent transplantation, without the need of enzymatic detachment [16–19]. Administration of CFSE-stained and microcarrier-attached keratinocytes and melanocytes in the *ex vivo* wound healing model would enable investigation of transplanted cells and their contribution to re-epithelialisation in a standardised and reproducible way.

The aim of the present study was to investigate whether CFSE-staining affects viability, migration, and proliferation during *in vitro* culture of keratinocytes and melanocytes. Moreover, the possible tracking of CFSE-stained cells and their contribution to the re-epithelialisation process following transplantation to human *ex vivo* wounds was evaluated.

## Methods

### Cell and tissue culture

Keratinocytes and melanocytes were isolated from full thickness skin biopsies obtained from healthy female patients (aged between 18 and 55) undergoing routine mammoplasty surgeries performed at the department of Hand Surgery, Plastic Surgery and Burns, Linköping University Hospital. The tissue was discarded and deidentified in accordance to ethical guidelines at Linköping university hospital. The study was performed with ethical permission from the Swedish Ethical Review Authority (protocol no. 2018/97-31). Informed oral consent was obtained from all patients. Primary cells were isolated using enzymatic digestion as previously described [20, 21]. Keratinocytes were expanded and cultured throughout experiments in keratinocyte serum free medium (KSFM). Melanocytes were expanded and cultured in melanocyte growth medium (MGM) (Table 1). Media was changed every second day.

**Table 1:** Cell culture media composition and suppliers.

Medium	Abbreviation	Base	Supplements	Manufacturer
**Keratinocyte serum free medium**	KSFM	Serum free medium with L-glutamine	25 μg/mL bovine pituitary extract 1 ng/mL epidermal growth factor 50 U/mL penicillin 50 μg/mL streptomycin	Gibco, Thermo Fisher Scientific
**Melanocyte growth medium**	MGM	PC-1 base medium	2% PC-1 supplement 1% L-glutamine 5 ng/mL basic fibroblast growth factor 24.6 g/mL N6,2′-O-Dibutyryl-adenosine 3′,5′-cyclic monophosphate 50 U/mL penicillin 50 μg/mL streptomycin	Base: Lonza Supplements: Sigma-Aldrich
**Fibroblast medium**	FM	Dulbecco’s modified Eagle medium	10% foetal calf serum 50 U/mL penicillin 50 μg/mL streptomycin	Gibco, Thermo Fisher Scientific

Cells were seeded and cultured on CultiSpher-S microcarriers (PerCell Biolytica, Åstorp, Sweden) as previously described [18]. Briefly, cells were enzymatically detached, mixed with hydrated microcarriers (50,000 cells/2 mg microcarriers/ml culture medium), and left to attach under intermittent agitation (5 min/hour) in spinner flasks. After 24 hours, agitation was set to constant to facilitate oxygen and nutrient exchange and the amount of KSFM or MGM was doubled, resulting in a microcarrier concentration of 1 mg/ml in solution. Approximately half of the culture medium was changed every second day.

*Ex vivo* wounds were prepared from viable human skin obtained from mammoplasty surgery as discarded and deidentified tissue in accordance to ethical guidelines at Linköping university hospital as previously described [15]. In brief, circular discs of the skin sample were excised using a 6 mm skin biopsy punch, and on the epidermal side in the centre of each disc a deep dermal wound was created with a smaller biopsy punch (3 mm wide and approximately 1 mm deep). The *ex vivo* wounds were cultured in fibroblast medium (FM) (Table 1) and culture medium was changed every second day.

### Staining efficiency

Staining efficiency of primary keratinocytes and melanocytes subjected to CFSE was investigated by culturing cells on chamber slides and adding 5 μM CFSE (CellTrace CFSE Cell Proliferation Kit, Molecular Probes Invitrogen, Life Technologies, Waltham, MA) according to manufacturer’s recommendation. 24 hours following staining, slides were mounted using Prolong Gold containing 4',6-diamidino-2-phenylindole (DAPI) (Molecular Probes). Three high power fields at 20x magnification were captured using an Olympus BX41 fluorescence microscope (Olympus Corporation, Japan), and overlay images of CFSE stain and nuclear DAPI stain were constructed in Photoshop CS6 (Adobe, San Jose, CA). Numbers of stained and non-stained cells were counted using FIJI (National Institutes of Health, Bethesda, MD) [22], averaged over three high power fields and reported as percentage stained cells of total amount of cells.

### Viability and migration of CFSE-stained keratinocytes and melanocytes

The viability of CFSE-stained keratinocytes and melanocytes was investigated using a (4, 5-dimethylthiazol-2-yl)-2, 5-diphenyltetrazolium bromide (MTT) assay at four time points; 24 hours, 48 hours, 72 hours and seven days. Keratinocytes were seeded at 45,000 cells/ml in flat 96-well plates in KSFM. Melanocytes were seeded at 50,000 cells/ml in flat 96-well plates in MGM. Two concentrations of CFSE were investigated: 5 μM and 10 μM. CFSE was diluted in pre-heated (37° C) PBS to the specified concentrations, added to wells and incubated for 15 minutes at 37° C. After removal of CFSE, keratinocytes and melanocytes were incubated in pre-heated (37° C) KSFM or MGM for 30 minutes. Medium was changed after 30 minutes and cells were cultured for set times. At the above-mentioned time points, medium was removed and replaced with 3 mg/ml MTT (Sigma-Aldrich) followed by four hours incubation at 37° C. MTT was removed and cells were incubated with DMSO for 10 minutes at 37° C. Absorbance was measured at 570 nm using a Versamax microplate reader (Molecular Devices, Sunnyvale, CA). Viability was reported as percentage relative to unstained controls. A two-way ANOVA with a Bonferroni’s multiple comparisons test was performed using Prism v7.0 (GraphPad software, La Jolla, CA) to test for differences in viability following staining with CFSE in respective cell types. *P* < 0.05 was considered statistically significant.

For the migration assay, keratinocytes and melanocytes were allowed to adhere and form a confluent layer in six-well plates. Cells were incubated for two hours at 37° C with 10 μg/ml of mitomycin C (Roche Diagnostics, Basel, Switzerland) to inhibit proliferation. Mitomycin was removed and cells washed twice in pre-heated PBS. CFSE was prepared to a concentration of 5 μM in PBS and cells were stained as previously stated. A scratch was inflicted across the centre of each well using a p200 pipette tip and images representing time point zero were captured. The scratches were monitored after three, eight and 24 hours. The remaining empty areas were traced and measured using FIJI and compared to time point zero, rendering a percentage of covered area at each time point. Three assays for each cell type were performed and significance was investigated using Prism v7.0 (Graphpad Software), with a two-way ANOVA repeated measurements and a Bonferroni's multiple comparisons test. *P* < 0.05 was considered significant.

### Proliferation of CFSE-stained keratinocytes and melanocytes on microcarriers

Attachment of CFSE-stained keratinocytes and melanocytes to CultiSpher-S microcarriers (PerCell Biolytica) was investigated by monitoring samples with fluorescence microscopy after 24 hours as well as MTT viability assays after 24 and 72 hours. Samples were removed from spinner flasks and directly investigated using a fluorescence microscope to observe CFSE-staining of cells adherent to microcarriers. Samples intended for viability staining were left to sediment and the supernatant was replaced with a matching volume of 3 mg/ml MTT-solution followed by 45-minute incubation at 37° C.

The proliferation of CFSE-stained keratinocytes and melanocytes was investigated using flow cytometric analysis of fluorescence intensity of CFSE. Cells were CFSE-stained as previously described and divided into spinner flask culture and adherent control cultures. A vial of sterilized 20 mg/ml microcarrier solution was left to sediment and the supernatant was removed. The stained cells were mixed with the microcarriers and added to a spinner flask under intermittent agitation (5 min/hour). After 24 hours, agitation was set to constant and the amount of KSFM or MGM was doubled, resulting in a microcarrier concentration of 1 mg/ml in solution. Proliferation was investigated after three days of culture. Samples corresponding to 24 mg of microcarriers were removed from the spinner flask at set time points, and enzymatically digested using trypsin at 37° C for two minutes and vortexed. Cells were passed through a filter (30 or 10 μm pore size for keratinocytes and melanocytes, respectively) (Celltrics, Partec GmbH, Görlitz, Germany). Cells were washed in FM and in PBS, and passed through respective filters before measurements. Adherent keratinocyte and melanocyte cultures were detached using trypsin, washed, filtered, and stained with CFSE according to above. Measurements at 488 nm excitation were performed using a Gallios flow cytometer (Beckman Coulter Inc., Brea, CA).

Analysis was performed using Kaluza software (Beckman Coulter). The percentage of divided cells in microcarrier culture was compared to adherent controls for each cell type and groups were compared using a Student’s *t*-test in Prism v7.0 (Graphpad Software). *P* < 0.05 was considered significant.

### Transplantation of CFSE-stained keratinocyte suspension to ex vivo wounds

Adherent keratinocytes were stained with 5 μM CFSE as previously described, trypsinised, washed in FM and resuspended in KSFM. Keratinocytes were pipetted directly to *ex vivo* wounds at a concentration of 90,000 cells/ml. FM was added up to the wound edge and the wounds were submerged in FM after 24 hours. Wounds were cultured for seven days in individual 24-well plate wells.

### Transplantation of CFSE-stained keratinocytes and CFSE-stained melanocytes to ex vivo wounds with the aid of microcarriers

Tissue culture of *ex vivo* wounds with added microcarriers with CFSE-stained keratinocytes and CFSE-stained melanocytes was performed. The re-epithelialisation and localisation of transplanted cells were monitored after seven, 14 and 21 days. The microcarriers with CFSE-stained keratinocytes and microcarriers with CFSE-stained melanocytes, respectively, were removed from the spinner flasks, left to sediment and were administered to wounds. Wounds were placed in individual wells in 24-well plates and FM was added up to the wound edge. After 24 hours, additional FM was added and the wounds were cultured submerged in medium for the remainder of the experiment. Control wounds were cultured in FM for corresponding times without microcarriers or cells.

### Co-cultivation and transplantation of CFSE-stained keratinocytes and CFSE-stained melanocytes

Cells were stained with 5 μM CFSE as previously described. Equal amounts of CFSE-stained keratinocytes and melanocytes were mixed with sterile Cultispher-S microcarriers and cultured in spinner flasks as previously described with 50% KSFM and 50% MGM and a final cell concentration of 90,000 cells/ml. Microcarriers with both cell types were deposited to *ex vivo* wounds and analysed after seven, 14 and 21 days of culture.

### Sectioning, fixation and immunohistochemical staining

Wounds were harvested at set time points, attached to sample holders using O.C.T cryomount medium (HistoLab, Gothenburg, Sweden) and snap-frozen in liquid nitrogen. Wounds were sectioned using a Leica CM 3050 cryostat (Leica Microsystems, Wetzlar, Germany) and mounted on Superfrost plus glass slides (Thermo Fisher Scientific, Waltham, MA).

Sections of wounds with transplanted keratinocyte suspension, and keratinocytes or melanocytes on microcarriers were fixed with 4% paraformaldehyde (PFA) for 10 minutes followed by washing in PBS and mounting with Prolong Gold mounting media containing DAPI (Molecular Probes). Slides were investigated using an BX41 fluorescence/light microscope and images captured with a DP70 camera (Olympus Corporation, Tokyo, Japan).

Sections of wounds with added microcarriers with keratinocytes only or co-cultivated keratinocytes and melanocytes intended for immunohistochemical staining were fixed for 15 minutes in 4% PFA followed by washing in PBS. The samples were blocked for 30 minutes in a humidified chamber with 2.5% bovine serum albumin followed by wash in PBS. Slides were drained and incubated for 60 minutes with primary antibodies (anti-Cytokeratin AE1/AE3, MAB3412, Merck Millipore, Darmstadt, Germany) at 1:200 dilution. Subsequently, slides were washed in PBS and incubated for 60 minutes with secondary antibodies (Alexa Fluor® 546, Molecular probes) at 1:500 dilution. Slides were mounted with Prolong Gold mounting media (Molecular Probes) and investigated using a fluorescence microscope.

## Results

**Fig 1:**
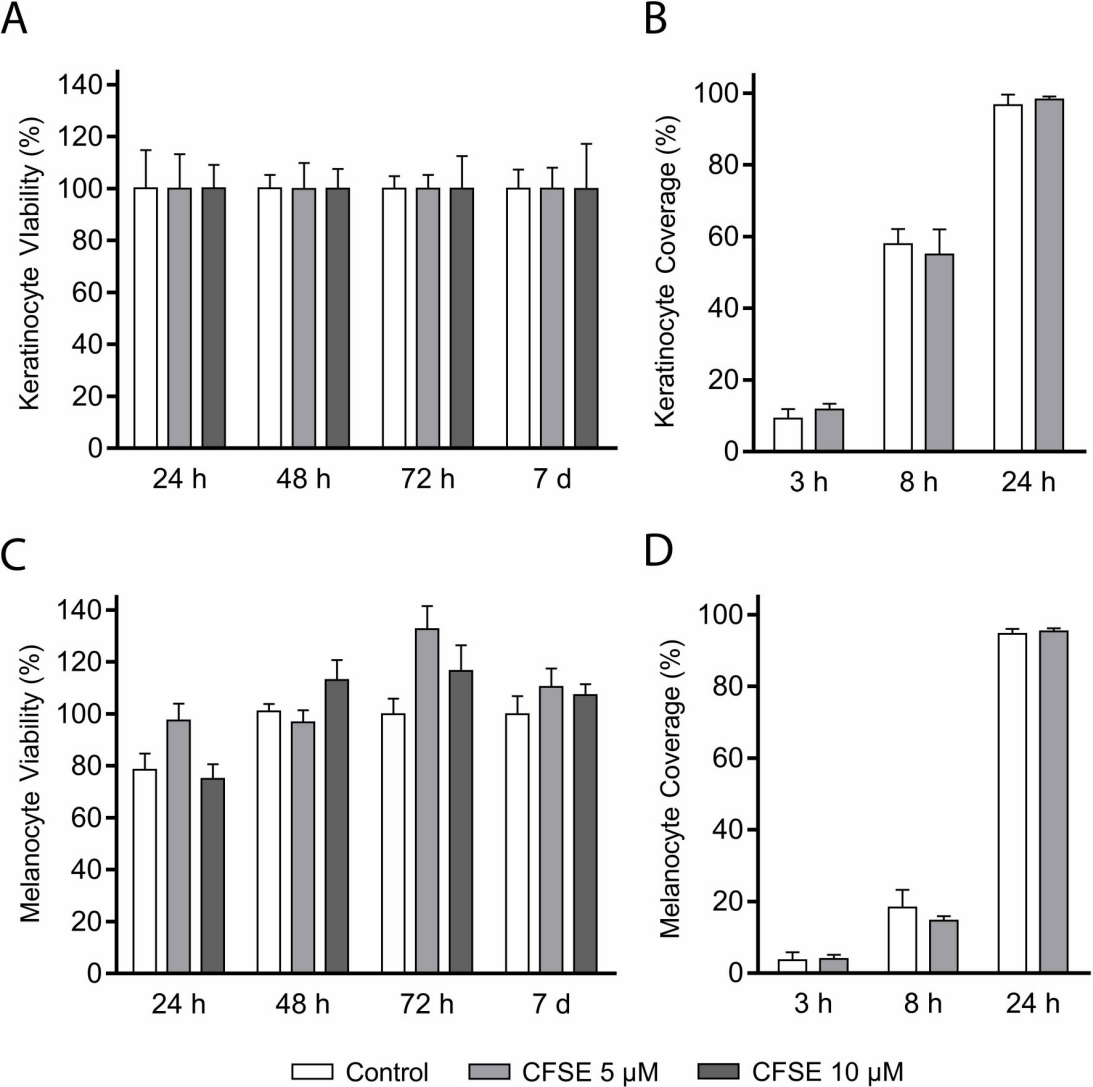
Viability and migration of CFSE-stained keratinocytes and melanocytes. The 5(6) carboxyfluorescein-N-hydroxysuccinimidyl ester (CFSE) diffuses over the cell membrane and is trapped inside the cell when its acetate groups are cleaved by intracellular esterases. Cleavage yields a green fluorescence. Tested concentrations of CFSE did not significantly reduce viability (A) or migration (B) of keratinocytes, nor the viability (C) or migration (D) of melanocytes. Viability values are relative to the unstained control and reported in percentage (mean±SD, *p*<0.05, n = 6). Results from the migration scratch assay are reported as coverage of the initial cell free area in percentage (mean±SD, *p*<0.05, n = 6).

Viability of keratinocytes stained with 5 μM or 10 μM CFSE was not significantly reduced at any of the investigated time points (Fig 1A, Table 2). Subsequent migration analysis of keratinocytes stained with 5 μM CFSE revealed no effect on the capacity to migrate (Fig 1B). Viability of CFSE-stained melanocytes was not significantly reduced, yet measured values were lower for melanocytes stained with 10 μM CFSE at 24 hours, and variance was larger compared to values for CFSE-stained keratinocytes (Fig 1C, Table 2). The ability for melanocytes to migrate was not affected by 5 μM CFSE-staining (Fig 1D).

**Table 2:** Viability measurements using (4, 5-dimethylthiazol-2-yl)-2, 5-diphenyltetrazolium bromide (MTT) assay of primary keratinocytes and melanocytes stained with 5(6) carboxyfluorescein-N-hydroxysuccinimidyl ester (CFSE). All values reported as percent relative to unstained controls (at time 0) ± SD, with Bonferroni’s multiple comparisons test corrected *p*-values.

**Keratinocytes**				
**CFSE**	**24 hours**	**48 hours**	**72 hours**	**7 days**
**0 μM**	100.2±36.1	100.2±12.6	100.0±11.9	100.0±18.2
**5 μM**	88.6±18.4 *p =* 0.720	99.83±24.7 *p*>0.999	100.0±12.9 *p*>0.999	100.0±19.9 p>0.999
**10 μM**	100.2±22.0 *p*>0.999	100.0±18.7 *p*>0.999	88.2±12.1 *p =* 0.670	85.2±26.1 *p =* 0.484
**Melanocytes**				
**CFSE**	**24 hours**	**48 hours**	**72 hours**	**7 days**
**0 μM**	78.5±15.2	101.0±5.6	99.8±14.9	99.8±17.4
**5 μM**	97.5±16.0 *p* = 0.133	96.8±9.5 *p*>0.999	132.6±20.0 *p* = 0.004	114.0±19.0 *p* = 0.839
**10 μM**	75.0±13.9 *p*>0.999	113.0± 15.6 *p* = 0.879	116.5±24.2 *p* = 0.230	107.2±9.5 *p*>0.999

**Fig 2:**
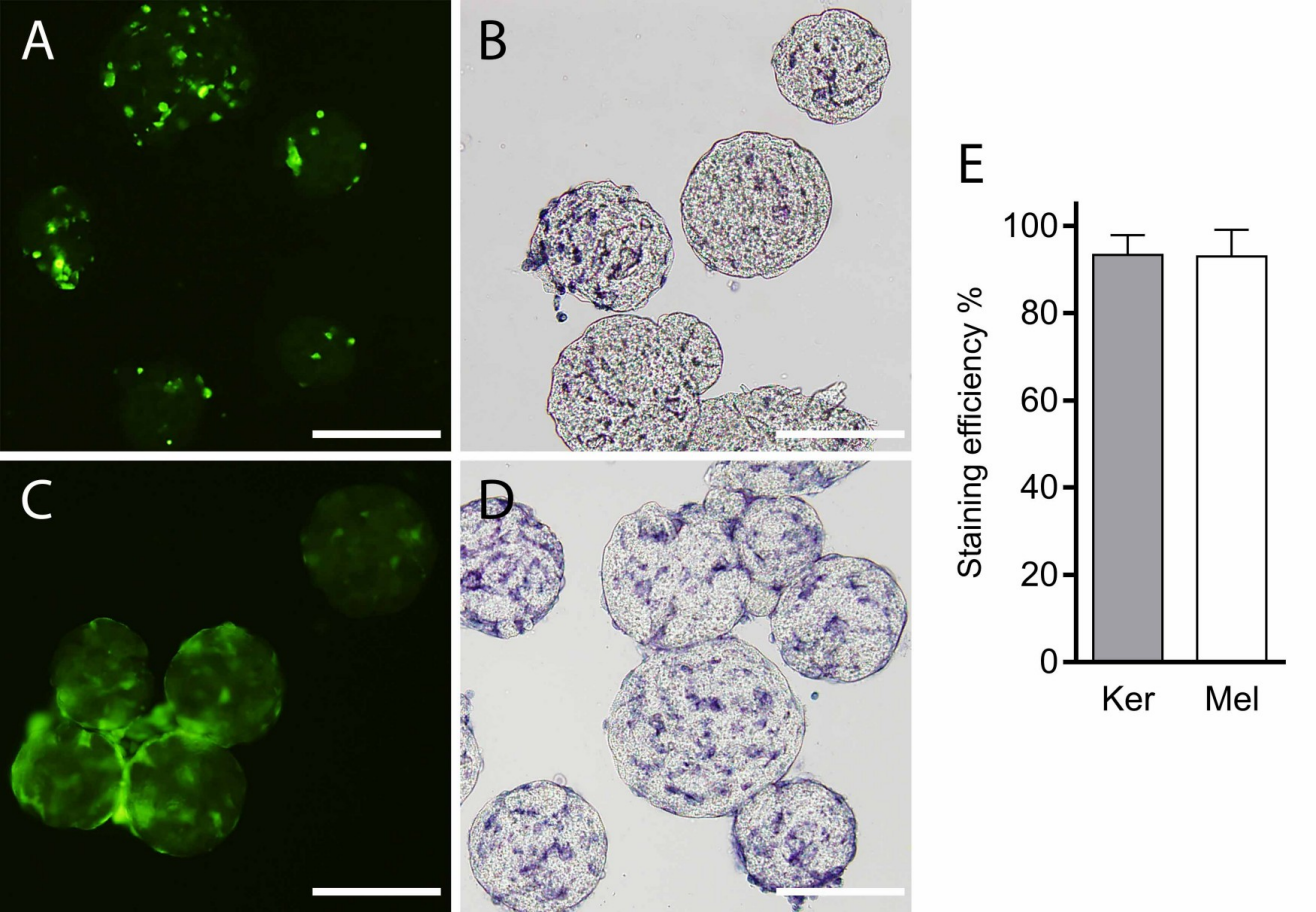
Staining efficiency and microcarrier attachment. (A) Keratinocytes stained with 5 μM 5(6) carboxyfluorescein-N-hydroxysuccinimidyl ester (CFSE) after 24 hours in spinner flask culture. (B) (4, 5-dimethylthiazol-2-yl)-2, 5-diphenyltetrazolium bromide (MTT)-staining of CFSE-stained keratinocytes on microcarriers after 72 hours in spinner flask culture, indicating viable cells. (C) Melanocytes stained with 5 μM CFSE after 48 hours in spinner flask culture. Scale bar = 200 μm (D) MTT-staining of melanocytes on microcarriers after 72 hours in spinner flask culture, indicating viable cells. In both the CFSE and MTT stainings, cells occupying outer surface as well as the inner pores of the microcarriers can be observed. (E) Staining efficiency of 5 μM CFSE investigated 24 hours after staining; 93.3 ± 2.6% of keratinocytes and 93.0 ± 3.5% of melanocytes were positive for CFSE and nuclear staining (mean ± SD, n = 3). Ker = keratinocytes; Mel = melanocytes. Scale bars = 300 μm.

**Fig 3:**
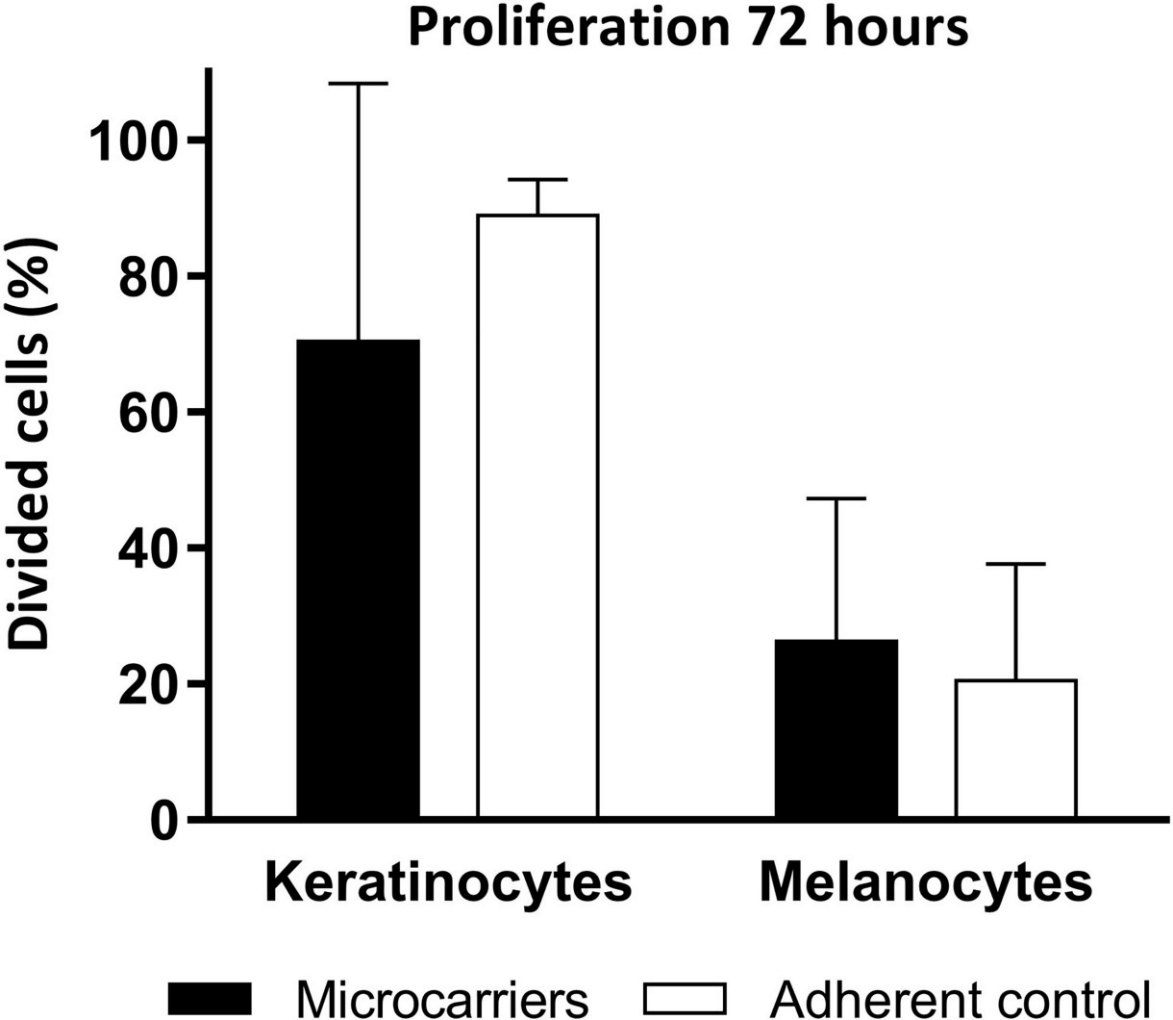
Proliferation of CFSE-stained keratinocytes and melanocytes on microcarriers. Flow cytometric analyses of 5(6) carboxyfluorescein-N-hydroxysuccinimidyl ester (CFSE)-staining in keratinocytes and melanocytes cultured on microcarriers in spinner flask culture and on cell culture polystyrene. No significant difference in percentage of divided cells was found between the culture methods (mean ± SD, *p*>0.05, n = 3).

Staining efficiency of primary keratinocytes and melanocytes was similar, 93.0% for keratinocytes, 93.3% for melanocytes and CFSE-staining did not affect attachment of either cell type to microcarriers (Fig 2). Proliferation of either cell type was not affected significantly when cultured on the microcarrier scaffolds compared to adherent controls on cell culture polystyrene (keratinocytes on microcarriers 70.4±37.9%, adherent culture 88.8±5.4%; melanocytes on microcarriers 26.3±21.0%, adherent culture 20.46±17.2%, all reported as mean ± SD) (Fig 3). The fluorescence intensity of the six-hour CFSE-control was subtracted from the intensity values of the investigated cultures during the analysis to obtain the reported percentage of divided cells.

**Fig 4:**
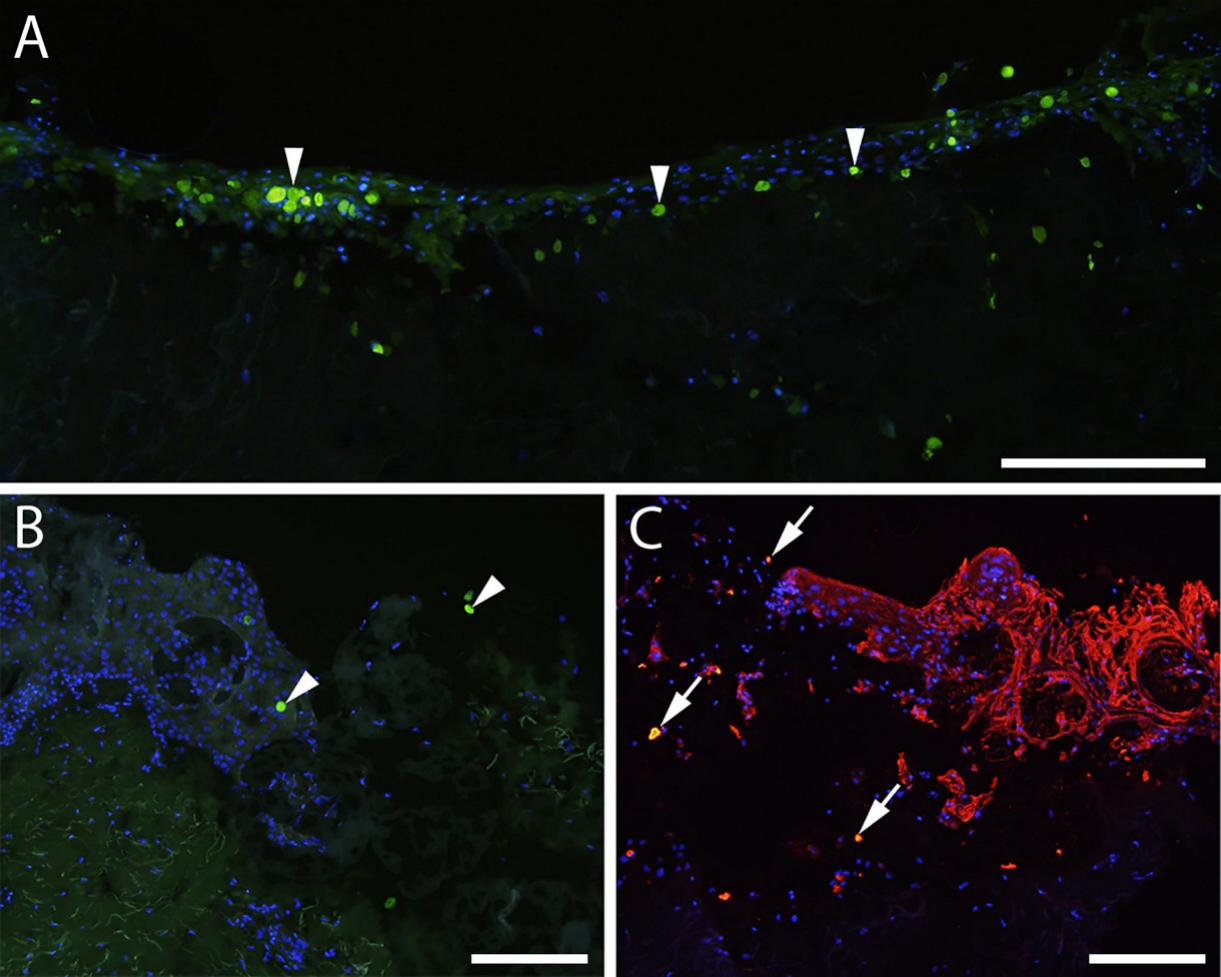
Transplantation of CFSE-stained keratinocytes to ex vivo wounds. (A) 5(6) carboxyfluorescein-N-hydroxysuccinimidyl ester (CFSE)-stained keratinocytes are incorporated into the neoepidermis in a fully re-epithelialized *ex vivo* wound seven days post transplantation, seen as green stained cells. (B) CFSE-stained keratinocytes (green) on microcarriers in an *ex vivo* wound seven days post transplantation. Arrowheads indicate CFSE-stained keratinocytes. (C) CFSE-stained keratinocytes on microcarriers in an ex vivo wound 21 days post transplantation. Double staining with CFSE and antibodies against cytokeratin detected with red fluorescent secondary antibodies can be identified by the yellow color (arrows), confirming that the stained cells are transplanted keratinocytes. Scale bars = 200 μm.

Keratinocytes stained with 5 μM were transplanted to *ex vivo* wounds in suspension. The presence of CFSE-stained keratinocytes in tissue sections was investigated using fluorescence microscopy. Green fluorescent cells (Fig 4A) could be detected at all time-points, and incorporation of the stained cells in the neoepithelial outgrowth was evident.

CFSE-stained keratinocytes were transplanted to *ex vivo* wounds attached to microcarriers and investigated for green fluorescent cells (Fig 3B) or double-labelled keratinocytes stained with both CFSE and pancytokeratin antibodies (Fig 3C). Transplanted keratinocytes were detected in wounds with both approaches and at all time-points.

**Fig 5:**
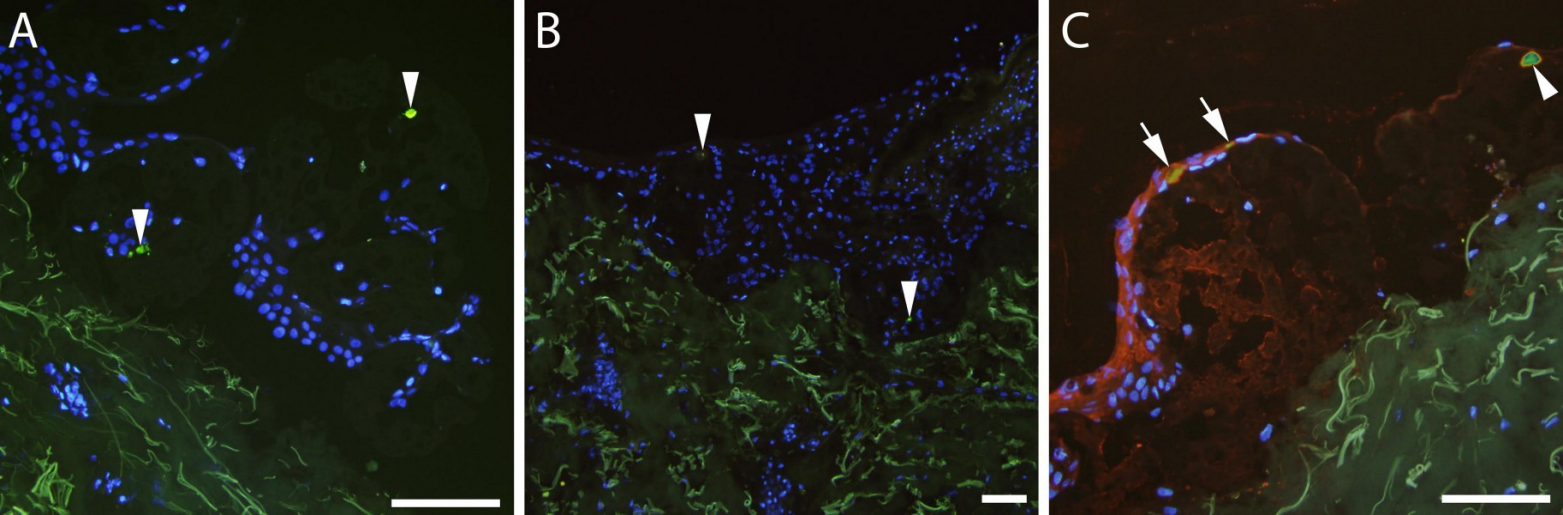
Transplantation of CFSE-stained melanocytes to ex vivo wounds. 5(6) carboxyfluorescein-N-hydroxysuccinimidyl ester (CFSE)-stained melanocytes (arrowheads) on microcarriers in an *ex vivo* wound seven (A) and 14 (B) days post transplantation (green staining). (C) Wound edge seven days post transplantation of co-cultured CFSE-stained keratinocytes and melanocytes. Sections were stained with keratinocyte-specific cytokeratin antibodies; double stained keratinocytes are indicated by arrows, green fluorescent melanocytes by arrowheads. Scale bars = 100 μm.

When attached to microcarriers and transplanted to *ex vivo* wounds, CFSE-stained melanocytes were detected in tissue sections from all time points. Representative images show green fluorescent cells on microcarriers (Fig 5A) and incorporated in a re-epithelialised wound (Fig 5B) after two weeks in culture.

CFSE-stained melanocytes and keratinocytes were co-cultivated in spinner culture and transplanted to *ex vivo* wounds. Sections were stained with antibodies against pancytokeratin to obtain double-labelled keratinocytes and green fluorescent melanocytes (Fig 5C). Both cell types were present in sections from all time points, and the two cell types were distinguishable in the immunohistochemically stained sections.

## Discussion

The present study was performed to elucidate the effects of CFSE-staining on primary keratinocytes and melanocytes, and to investigate the possibility to track transplanted cells in skin tissue sections with the aid of CFSE-staining. CFSE was originally developed in order to investigate lymphocyte migration [23] and commonly used today in studies on proliferation of lymphocytes or other blood cells [24, 25]. The present study is the first example of using CFSE to track keratinocytes and melanocytes during cutaneous wound healing. Previous studies where CFSE-staining has been performed on primary keratinocytes [26, 27] and retinal pigment epithelium [14] have been published. The staining has been utilized as a proliferation marker, however no reports exist on either migration assays or viability measurements where CFSE-stained keratinocytes are compared to unstained control keratinocytes. To our knowledge only two previous studies where human melanocytes are stained with CFSE are present [28, 29]. Previous studies show that 5 μM CFSE-staining has been measurable with flow cytometry up to 14 days in keratinocytes [13], and our findings provide evidence that cells can be tracked for three weeks in an *ex vivo* wound healing model. Further studies are needed to assess the potential of using CFSE-staining to evaluate proliferation *in vivo*, potentially by capturing 3D tissue images using confocal fluorescence microscopy, or by measuring total fluorescence intensity in whole tissue.

Moreover, paraffin embedded, sectioned and fixated hybridomas have retained CFSE-staining after three weeks in culture indicating that standard histological processing should in fact not interfere with the fluorescent signal from CFSE [12], thus extending the usefulness of CFSE-staining for wound healing applications.

To exclude possible negative effects of staining with CFSE we investigated the viability and migration of CFSE-stained keratinocytes and CFSE-stained melanocytes. No significant negative effect on viability of keratinocytes or melanocytes could be observed at any of the time points. Cell culture with CFSE resulted in very stable values of the MTT-assay of the keratinocyte cultures, but more varying results for the melanocyte cultures. Primary melanocytes in culture are sensitive to handling and are preferably used at passages three or four, which could explain the difference in viability between the two cell types, as well as the increase in viability observed in the melanocyte culture after 72 hours. Based on the findings on viability of CFSE-stained cells, the choice of concentration for subsequent experiments was 5 μM. The migration capacity of keratinocytes and melanocytes stained with 5 μM CFSE was maintained at all time points. Taken together, results of the viability and migration assays indicate that staining with CFSE does not impair viability or normal migrational function of keratinocytes or melanocytes.

CFSE-stained keratinocytes were shown to retain their proliferation rates compared to keratinocytes cultured adherently on polystyrene but showed greater variability on the microcarriers than in 2D culture. No inhibition of proliferation could be observed when comparing melanocytes cultured on microcarriers to cells cultivated adherently on polystyrene. Retained proliferation of melanocytes attached to microcarriers has previously been shown in a study by Smit *et al*. where melanocytes cultured on collagen-coated microcarriers was shown to have a 50% higher proliferation rate compared to 2D culture [30]. As gelatine is a derivative of collagen, the properties of the materials may be similar. We could not find a significant increase in proliferation, but a retained ability to proliferate compared to adherent culture and an indication that the proliferation rate was higher on microcarriers in spinner flask culture. The findings on retained proliferation rates are of importance since proliferation on the microcarriers is crucial in order for microcarrier scaffolds to be used clinically. Previous studies have evaluated the clinical use of the gelatin microcarriers, illustrating good biocompatibility, absence of capsular formation, rejection or other adverse events [31]. Thus, the results from the present study illustrate that expansion of primary keratinocytes and melanocytes can be performed in spinner flask culture for both cell types, in both single or co-cultivation.

*Ex vivo* wounds with transplanted cells in suspension or on microcarriers were investigated at seven, 14 and 21 days post transplantation. Transplanted cells could clearly be detected in the tissue sections at all time points. This indicates that the CFSE-stain is suitable for cryosectioning applications and can withstand the processing with snap freezing and sectioning, in line with results presented by Bronner-Fraser [12]. Moreover, being fluorescein-based, CFSE is compatible with a range of fluorochromes and thus suitable for multicolor flow cytometry, enabling further applications in conjunction with proliferation measurements and cell tracking. Re-epithelialisation of *ex vivo* wounds without added microcarriers normally occurs in seven to ten days. The addition of CFSE-stained keratinocytes in suspension did not delay this process, as evidenced by fully re-epithelialised wounds at day seven, with transplanted cells incorporated into the neoepidermis covering the wound bed. Wounds transplanted with microcarriers and keratinocytes, or co-cultured keratinocytes and melanocytes were stained with antibodies against cytokeratin, which enabled distinction of the CFSE-stained cells and transplanted keratinocytes from wound edge cells migrating into the healing wounds. The application of CFSE-staining did not restrict the use of immunohistochemical staining on the tissue sections.

In this study, we have investigated the use of CFSE-staining for tracking transplanted cells during wound healing. The use of CFSE provides a simple passive staining of cells that is detectable for up to three weeks, requires no custom preparation of cells that are to be transplanted, and do not affect viability, migration or proliferation of either keratinocytes or melanocytes in cell culture or in an *ex vivo* wound healing model. Importantly, cells are not subjected to genetic alteration which may render them unsuitable for therapeutic use. We conclude that CFSE-staining is a promising way of tracking cells in skin transplantation studies, as well as in the evaluation of novel cell therapy-based treatments.

### Limitations

The current study tracks transplanted cells for three weeks in an *ex vivo* wound healing model. This model system contains all the local cells and mediators involved in wound healing, but not the systemic response. Thus, no or very little inflammatory response is present. This is a clear limitation, and the model is suitable for early evaluation as an intermediary step before performing animal or human experiments. Future studies will focus on autologous transplantation into immune-competent hosts, with extended follow-up time. Moreover, rapid cell division will result in dilution of the intracellular CFSE staining, and ultimately prevent detection. This is a consideration that needs to be taken depending on the cell type studied.
